# Effects of salt substitute on urinary electrolytes and blood pressure in a real-world setting—cohort study in Hunan, China

**DOI:** 10.3389/fnut.2024.1504152

**Published:** 2024-12-18

**Authors:** Hao Wu, Wenbin Ouyang, Jing Deng, Yongmei He, Lu Yin, Xia Cao, Zhiheng Chen, Pingting Yang, Yaqin Wang, Ying Li, Xin Huang

**Affiliations:** ^1^Department of Health Management, The Third Xiangya Hospital, Central South University, Changsha, China; ^2^Department of Epidemiology, Hunan Normal University School of Medicine, Changsha, China; ^3^Department of Epidemiology, Xiangya School of Public Health, Central South University, Changsha, China; ^4^Department of Health Management, Aerospace Center Hospital, Beijing, China; ^5^Medical Research & Biometrics Center, National Center for Cardiovascular Diseases, Chinese Academy of Medical Sciences and Peking Union Medical College, Beijing, China

**Keywords:** salt substitute, real-world, salt restriction, cohort, China

## Abstract

**Background and aims:**

Salt substitute is considered an effective strategy to reduce sodium and increase potassium intake and thereby lower blood pressure in China, but its benefits and risks are uncertain in real-world data. This study is designed to compare the difference in the 1-year efficacy of salt substitute and salt restriction on urinary electrolytes and blood pressure.

**Methods and results:**

A total of 2,929 and 2,071 participants with the 24-h estimated urinary sodium excretion (eUNaE) above 2.36 g/d using salt substitute (SS) and salt restriction (SR) strategies, respectively, were followed for 1 year. Salt substitute users were further divided by potassium chloride (KCl) content (13% vs 25%) and duration (9–11 vs 12 months). The 24-h eUNaE and estimated urinary potassium excretion (eUKE) levels were calculated using the Kawasaki formula from spot urine sample. The SS group (*n* = 1,897) had lower eUNaE (3.82 ± 1.03 vs 4.05 ± 1.01 g/day, *p* < 0.01) than the SR group (*n* = 1,897) after 1 year. Both 13 and 25% KCl substitutes reduced eUNaE versus restriction (*p* < 0.05). The SS group had a higher eUKE than the SR group (2.09 ± 0.43 vs 1.71 ± 0.62 g/day, *p* < 0.01). The eUKE was higher with 25% versus 13% KCl substitutes, while the Na/K was lower with 25% versus 13% KCl substitutes (*p* < 0.05). No significant blood pressure differences occurred between the SS and SR groups (*p* > 0.05), whereas 25% KCl exposure was related to a lower level of SBP, regardless of whether it was compared with SR or 13% KCl.

**Conclusion:**

Compared with salt restriction, salt substitute results in more sodium reduction and greater potassium increase. In spite of this, it does not result in better control of blood pressure, especially for the group receiving 13% KCl.

## Introduction

Elevated dietary sodium consumption, as well as low levels of dietary potassium intake, is associated with hypertension, cardiovascular disease, and premature death (1–3). Sodium consumption in China is high. In recent years, we found high sodium and low potassium intake levels in the Hunan population based on spot urinary analysis (4).

There is empirical evidence that replacing sodium chloride with a potassium-enriched salt substitute can significantly reduce blood pressure. Recently, a randomized trial named the SSaSS study proved that salt substitute could reduce the rates of stroke, major cardiovascular events, and death compared with regular salt among persons who had a history of stroke or over 60 years old and had high blood pressure (5). Moreover, a cluster-randomized trial conducted in elderly care facilities showed that using salt substitute instead of regular salt can lower systolic blood pressure by 7.1 mmHg. In contrast, restricting the supply of regular salt had no effect on systolic blood pressure6. Meanwhile, no apparent serious adverse effects were found in the salt substitute group (5, 6). These results indicated that salt substitute hold great potential for the control of blood pressure and prevention of chronic disease.

However, most salt substitute studies have been carried out in rural or closed areas to reduce the possibility of participants consuming regular salt (5–7). Thus, these studies may maximize the effect of the salt substitute. In fact, data from the China Health and Nutrition Survey showed that the contribution of added salt to total sodium intake dropped from 81% in 1991 to 70% in 20,098. The proportion of sodium intake from packaged food is gradually increasing among the Chinese population, especially in urban areas. Additionally, the salt substitute commonly available on the market usually contains 13% potassium chloride, rather than the 25% cited in the abovementioned research. Therefore, it is essential to evaluate the public health effects of salt substitute in the real world for the general population.

In the current study, we conducted a prospective cohort study and compared the effects of salt substitute usage to salt restriction on urinary electrolytes and blood pressure in the real world of urban China.

## Methods

### Participants

**Figure 1:**
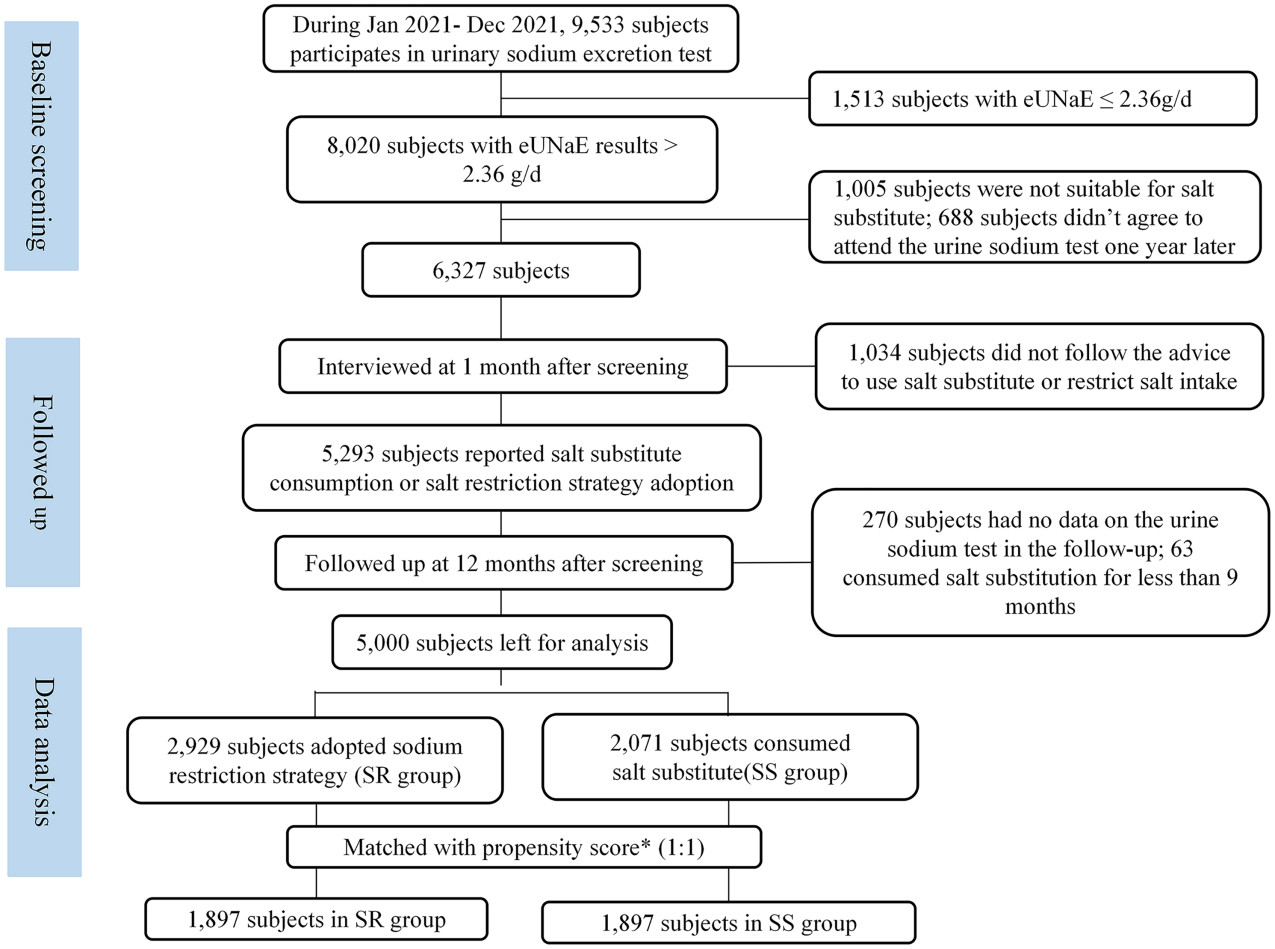
Propensity score matched subjects enrollment flowchart. Propensity score was calculated based on age, sex, BMI category, alcohol usage, smoking, hypertension, diabetes, dyslipidemia, level of eUNaE and eUKE at baseline screening. SR, salt restriction; SS, substitute salt.

The study was conducted at the Third Xiangya Hospital of Central South University in Changsha, a city in South China. All the subjects participating in annual health check-ups between January 2021 and December 2021 who had the 24-h estimated urinary sodium excretion (eUNaE) exceeding 2.36 grams/day (approximately equivalent to salt intake above 6 grams/day, which surpasses the recommended level stipulated by the Chinese Dietary Guidelines) and were advised by their physicians to reduce sodium intake. The suggestions included raising awareness about salt reduction, reducing dining out, reading food nutrition labels, decreasing the intake of high-salt foods, changing cooking methods, recommending the use of measuring tools such as salt restriction spoons, and salt substitute. In the first month following baseline sodium screening, all selected subjects were interviewed by skilled physicians through telephone, and those who reported that they had already consumed salt substitute or adopted salt reduction strategy without salt substitute were enrolled in our study. Participants who met the following criteria were further excluded: aged < 18 years; not suitable for salt substitute usage, including those working in a high-temperature or labor-intensive environment, or having chronic kidney disease, stroke, coronary heart disease, or who took drugs that affect sodium and potassium excretion, such as ACEIs or ARBs and diuretics; implemented salt substitute before baseline screening or implemented salt substitute for less than 9 months during the follow-up period; neither followed the recommendation to replace salt with salt substitutes nor to restrict salt by consuming salt restriction spoons or salt-reduced seasonings; and did not agree to be followed and attended physical examination at 12 months after baseline screening. All participants provided written informed consent to participate in the study, and the enrollment process is described in Figure 1.

### Follow-up and exposure assessment

After baseline screening, the participants were followed up at months 1 and 12. At the first follow-up visit, the subjects were interviewed through telephone to determine their salt exposure. During follow-up visits at month 12, participants were required to attend the annual health check-up and to complete an online questionnaire regarding their salt consumption behavior, including whether they followed the physician’s recommendations to reduce salt intake, and avoid high salt foods, especially the duration of using SS. In this check-up, each participant had to report their medical visits and symptoms in the previous year and received urine sodium and potassium, serum potassium, and electrocardiogram tests.

In our study, all enrolled participants were advised by physicians to limit sodium intake by using salt substitute. Salt substitute, however, had to be purchased directly from the market by themselves. The Chinese market offers two types of salt substitute with different potassium chloride (KCl) contents. The main product contains 13% KCl, which contains 6,556 mg of potassium per 100 grams, whereas the other type of product contains 25% KCl, which contains 13,110 mg of potassium per 100 grams (these data may vary slightly among products from different manufacturers). For individuals suitable for salt substitute usage, physicians in our health management department only recommended the substitute strategy but not the type of product. Our study consisted of two groups of participants based on reported salt consumption: (1) The salt substitute (SS) group refers to participants who cook at home using salt substitutes they have already purchased; (2) the salt restriction (SR) group comprises those who did not use SS at any time point during follow up and had other salt restriction action such as consumed salt-restricted spoons. At the last follow-up, the cumulative duration of SS exposure was collected and those who used SS but had less than 9 months of exposure been excluded. Over 2,134 subjects reported exposure to SS and 97% of whom had been exposed for more than 9 months, and they could be further classified into two subgroups: 12 months and 9–11 months.

### Outcome and covariate measures

The National Physical Examination Questionnaire was used to collect sociodemographic characteristics, alcohol usage, cigarette smoking, and medical history. Physical examinations were conducted by trained physicians with the same methods described in our previous studies (4, 8–10). The definitions of chronic diseases are detailed in the references (11–16). According to the “Dietary Guidelines for Chinese Residents (2016) (13),” the recommended daily salt intake of Chinese adults over the age of 18 does not exceed 6 grams (approximately equivalent to the sodium intake not exceed 2.36 grams/day), and the daily potassium intake is more than 3.6 grams, respectively (17). Based on these reference amounts, we determined whether an individual’s intake of sodium and potassium met the recommended levels.

As described in our previous studies, fasting venous blood samples were collected and analyzed immediately at the clinical laboratory. Meanwhile, mid-stream urine samples were tested for sodium, potassium, and creatinine within 2 h of collection. Sodium and potassium were measured by the ion selective electrode method, and creatinine was measured by the dynamic enzymatic method. In the present study, 24-h urinary sodium (eUNaE) and potassium (eUKE) excretion were determined using the Kawasaki approach (18) as a proxy for average daily sodium and potassium intake. One gram of urine sodium is calculated to equal 2.55 grams of salt intake.

### Statistical analyses

It was determined that 5,283 subjects met the inclusion and exclusion criteria. Among them, 5,000 (94.64%) had complete data on the urine sodium test at the last follow-up and were analyzed. However, most of the medical characteristics at baseline were found to be significantly different between the SS and SR groups (Table 1), and a propensity score matching (PSM) approach was used to obtain comparable groups. In particular, a logistic regression model based on age, gender, BMI category, alcohol consumption, smoking, hypertension, diabetes, dyslipidemia, and levels of eUNaE and eUKE at baseline was applied to determine propensity scores for each subject. Using a caliper set at 0.02, participants in the SS group were matched with participants in the SR group using the nearest neighbor matching method. Finally, a total of 3,794 participants were successfully matched and used for effect and safety analysis. As shown in Table 1, the implementation of PSM significantly reduced baseline differences between SR and SS groups, with standardized mean differences (SMDs) for all covariates within 10%, indicating a high degree of baseline balance.

**Table 1:** Distribution of social demographic and clinical characteristics at baseline in unmatched overall and propensity score matched cohort.

Characteristics at baseline	Unmatched overall cohort				Propensity score matched cohort			
	SR (*n* = 2,929)	SS (*n* = 2,071)	SMD	*p*	SR (*n* = 1,897)	SS (*n* = 1,897)	SMD	*p*
Age (years), *n* (%)								
18–34	536 (18.30)	380 (18.35)	−0.01	0.73	346 (18.24)	354 (18.66)	0.01	0.90
35–49	1,466 (50.05)	10,415 (49.62)			936 (49.34)	923 (48.66)		
≥ 50	927 (31.65)	676 (32.64)			615 (32.42)	620 (32.68)		
Sex, *n* (%)								
Female	848 (28.95)	763 (36.84)	0.17	< 0.01	655 (34.53)	689 (36.32)	−0.04	0.25
Male	2081 (71.05)	1,308 (63.16)			1,242 (65.47)	1,208 (63.68)		
Alcohol usage, *n* (%)	1,656 (56.54)	1,223 (59.05)	0.06	0.08	1,019 (53.72)	1,058 (55.77)	0.03	0.20
Smoking, *n* (%)	903 (30.83)	525 (25.35)	0.12	< 0.01	441 (23.25)	420 (22.14)	0.01	0.42
BMI category, *n* (%)								
Underweight	53 (1.81)	42 (2.03)	0.11	< 0.01	46 (2.42)	36 (1.90)	0.02	0.56
Normal weight	1,261 (43.05)	1,001 (48.33)			888 (46.81)	920 (48.50)		
Overweight	1,286 (43.91)	827 (39.93)			779 (41.06)	759 (40.01)		
Obese	329 (11.23)	201 (9.71)			184 (9.70)	182 (9.59)		
SBP (mmHg), mean (SD)	120.90 (14.58)	121.12 (14.92)	0.03	0.60	120.42 (14.98)	120.80 (14.76)	−0.02	0.95
DBP (mmHg), mean (SD)	75.00 (10.69)	74.51 (10.77)	0.04	0.11	74.51 (10.76)	74.53 (10.79)	−0.00	0.43
Normal BP (mmHg), *n* (%)	2,569 (87.71)	1827 (88.22)	−0.01	0.59	1,678 (88.46)	1,668 (87.93)	−0.03	0.62
Hypertension, *n* (%)	458 (15.64)	297 (14.34)	0.05	0.21	282 (14.87)	280 (14.76)	0.01	0.93
Diabetes, *n* (%)	206 (7.03)	121 (5.84)	0.04	0.09	116 (6.11)	109 (5.75)	−0.03	0.63
Dyslipidemia, *n* (%)	1,064 (36.33)	679 (32.79)	0.08	0.01	621 (32.74)	617 (32.53)	−0.02	0.89
eUNaE (g/day), mean (SD)	4.43 (1.08)	4.51 (1.01)	−0.15	< 0.01	4.48 (1.12)	4.51 (1.01)	0.03	0.36
eUKE (g/day), mean (SD)	2.27 (0.47)	1.98 (0.54)	−0.51	< 0.01	2.11 (0.43)	2.08 (0.52)	−0.06	0.06
Na/K ratio, mean (SD)	2.03 (0.74)	2.57 (1.47)	0.51	< 0.01	2.21 (1.10)	2.28 (1.12)	0.07	0.06

SR, salt restriction; SS, salt substitute; SMD, standardized mean difference; SD, standard deviance; SBP, systolic blood pressure; DBP, diastolic blood pressure; BP, blood pressure; eUNaE, estimated urinary sodium excretions; eUKE, estimated urinary potassium excretions; BMI, body mass index.

Continuous variables are expressed as the means and standard deviation (SD), whereas categorical variables are expressed as counts (n) and percentages (%), and differences between the SS and SR groups were tested by ANOVA (analysis of variance) and the chi-square test, respectively. A change in eUNaE, eUKE, Na/K ratio, systolic blood pressure (SBP) or diastolic blood pressure (DBP) was calculated by subtracting baseline levels from 1 year after. The effects of salt substitute on eUNaE, eUKE, and the Na/K ratio 1 year after sodium restriction and changes in those three parameters were assessed using linear regression and expressed with standardized coefficients (*β*) and 95% confidence intervals (CIs). Meanwhile, the effects of using salt substitute on the risk of normal blood pressure (BP) and salt intake ≤ 6 g/d were assessed separately with logistic regression and expressed with odds ratios (ORs) and 95% CIs. Potential confounding variables in linear and logistic regression models included age (18–34, 35–49, and ≥ 50), sex (female or male), alcohol usage (yes or no), smoking (yes or no), BMI category (underweight, normal weight, overweight and obese), hypertension (yes or no), diabetes (yes or no), hyperglycemia (yes or no), and dyslipidemia (yes or no). We also analyzed the effect of SS in the unmatched cohort population, in populations with different exposure dosages and in populations with different cumulative exposure durations. The effect of SS among the hypertension subgroup was analyzed through stratification analysis. SAS version 9.4 (SAS Institute Inc.) was used for analyses.

## Results

### Characteristics of the enrolled participants at baseline

During 2021, 9,533 participants participated in sodium excretion screening, and of them, 8,020 participants showed eUNaE > 2.36 g/d, with a prevalence of 84.13% (95% CI: 83.38, 84.86%). A total of 5,000 participants met the inclusion criteria of our study and had tested sodium excretion at the 12-month follow-up. Among this unmatched population, participants in the SS group were more likely to be female, smokers, normal weight, diabetes, and dyslipidemia, with lower eUKE and higher eUNaE and Na/K ratios (*p* < 0.05) than participants in the SR group (Table 1). After PSM, these differences became insignificant (*p* > 0.05), and both groups were comparable in terms of those characteristics (Table 1). In the PS-matched SS group, 417 participants (21.98%) consumed SS with 25% KCl, and 264 participants (13.92%) started consuming SS between 1 and 3 months after using the SR strategy.

### Effects of SS on the level of eUNaE

**Figure 2:**
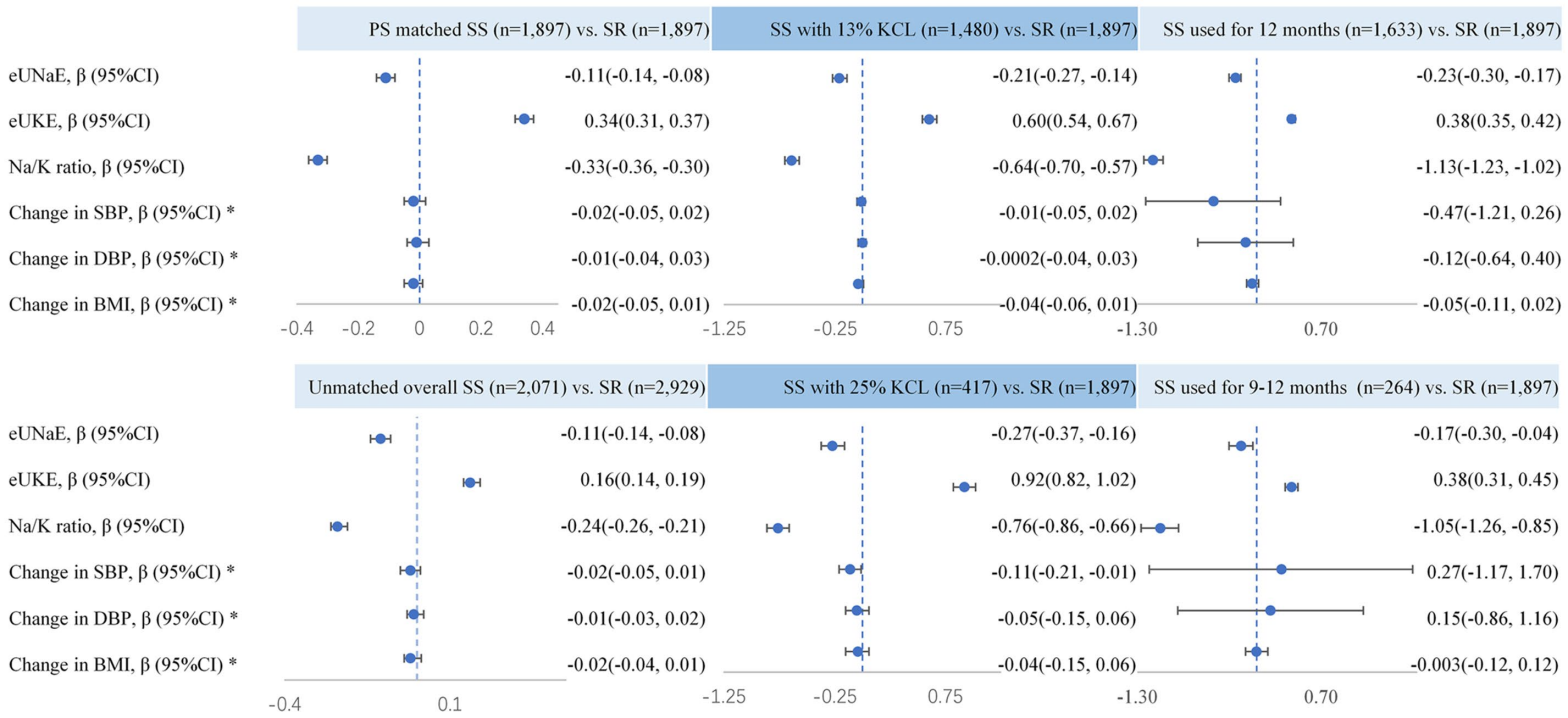
Effects of salt substitute usage compared with a regular salt restriction strategy among different exposure population. Changes in those indices were calculated by subtracting baseline levels from 1 year after and the effects of SS on those indices were adjusted for age, sex, alcohol usage and smoking, hypertension, diabetes, chronic gastritis, hyperglycemia, and dyslipidemia.

As shown in Table 2, compared to the baseline, the level of eUNaE decreased in both groups after 1 year of salt intake restriction, and the change level in the SS group was − 0.68 (SD 1.03) g/day, which was significantly greater than that in the SR group (−0.42 (SD 1.16) g/day) (*p* < 0.01). Therefore, the level of eUNaE 1 year later in the SS group (3.82 (SD 1.03) g/day) was substantially lower than that in the SR group (4.05 (SD 1.01) g/day) (*p* < 0.01), and the incidence of daily salt intake ≤ 6 g in the SS group [6.54% (95% CI: 5.47, 7.74%)] was higher than that in the SR group [3.43% (95% CI: 2.65, 4.35%)] (*p* < 0.01). After adjusting for confounding factors, SS was still significantly associated with a decreased level of eUNaE [*β* = −0.11, 95% (−0.14, −0.08)], a negative change in eUNaE [*β* = −0.12, 95% (−0.15, −0.09)] and a higher risk of salt intake ≤ 6 g/d [OR = 1.96, 95% (1.44, 2.67)] (Table 3). Compared to SR, either exposure to SS with 13% or 25% KCl or the use of SS for 12 months or 9–11 months may lead to lower levels of eUNaE (Figure 2; Supplementary Tables S1–S4). However, the eUNaE levels between the 13 and 25% KCl groups did not differ significantly (*p* > 0.05) (Supplementary Tables S1, S2). Using SS for 12 months or for 9–11 months also produced similar levels of eUNaE (Supplementary Tables S3, S4).

**Table 2:** Comparison on level of urinary sodium and potassium excretion, blood pressure, and BMI at 1 year after the implementation of different salt restriction strategies (*N* = 3,794).

Level of outcomes after 1 year of strategies implementation	SR (*n* = 1,897)	SS (*n* = 1,897)	*p*
eUNaE (g/day), mean (SD)	4.05 (1.01)	3.82 (1.03)	< 0.01
eUKE (g/day), mean (SD)	1.71 (0.62)	2.09 (0.43)	< 0.01
Na/K ratio, mean (SD)	3.02 (2.12)	1.90 (0.75)	< 0.01
Change in eUNaE (g/day)_*_, mean (SD)	−0.42 (1.16)	−0.68 (1.03)	< 0.01
Change in eUKE (g/day)^*^, mean (SD)	−0.40 (0.64)	0.01 (0.46)	< 0.01
Change in Na/K ratio^*^, mean (SD)	0.81 (2.09)	−0.38 (1.00)	< 0.01
BMI (kg/m2), mean (SD)	24.12(3.01)	24.14(3.07)	0.86
Change in BMI (kg/m2)*, mean (SD)	0.06 (0.95)	0.02 (0.95)	0.16
SBP (mmHg), mean (SD)	120.42 (14.98)	120.80 (14.76)	0.43
DBP (mmHg), mean (SD)	74.51 (10.76)	74.53 (10.79)	0.95
Change in SBP (mmHg)_*_, mean (SD)	−0.18 (11.42)	−0.51(11.33)	0.38
Change in DBP (mmHg)^*^, mean (SD)	−0.05 (8.09)	0.01 (7.87)	0.86
Salt intake ≤ 6g/d, *n* (%)	65 (3.43)	124 (6.54)	< 0.01
Potassium intake ≥ 3.6g/d, *n* (%)	0 (0.00)	1 (0.05)	0.32
Normal BP, *n* (%)	1,541 (81.23)	1,515 (79.86)	0.29

^*^Calculated by subtracting baseline levels from 1 year after.

SR, salt restriction; SS, salt substitute; SD, standard deviance; eUNaE, estimated urinary sodium excretions; eUKE, estimated urinary potassium excretions; SBP, systolic blood pressure; DBP, diastolic blood pressure; BP, blood pressure; BMI, body mass index.

**Table 3:** Effects of salt substitute usage on urinary sodium and potassium excretion, blood pressure, and BMI compared to a regular salt restriction strategy 1 year after implementation of the strategy (*N* = 3,794).

Outcome at 1 year after implementation of strategy	Model A┼			Model B╫			Model C╪		
	*β* ^#^	95%CI	*p*	*β* ^#^	95%CI	*p*	β^#^	95%CI	*p*
eUNaE (g/day)	−0.11	(−0.14, −0.08)	< 0.01	−0.11	(−0.14, −0.08)	< 0.01	−0.11	(−0.14, −0.08)	< 0.01
eUKE (g/day)	0.33	(0.30, 0.36)	< 0.01	0.34	(0.31, 0.37)	< 0.01	0.34	(0.31, 0.37)	< 0.01
Na/K ratio	−0.33	(−0.36, −0.30)	< 0.01	−0.33	(−0.36, −0.30)	< 0.01	−0.33	(−0.36, −0.30)	< 0.01
Change in eUNaE (g/day)^*^	−0.12	(−0.15, −0.09)	< 0.01	−0.12	(−0.15, −0.09)	< 0.01	−0.12	(−0.15, −0.09)	< 0.01
Change in eUKE (g/day)^*^	0.35	(0.32, 0.37)	< 0.01	0.34	(0.31, 0.37)	< 0.01	0.34	(0.31, 0.37)	< 0.01
Change in Na/K ratio (g/day)^*^	−0.34	(−0.37, −0.31)	< 0.01	−0.34	(−0.37, −0.31)	< 0.01	−0.34	(−0.37, −0.31)	< 0.01
DBP (mmHg)	0.001	(−0.03, 0.03)	0.95	0.01	(−0.02, 0.04)	0.66	0.01	(−0.02, 0.03)	0.68
SBP (mmHg)	0.01	(−0.02, 0.05)	0.43	0.02	(−0.01, 0.05)	0.25	0.02	(−0.01, 0.04)	0.24
Change in SBP (mmHg)^*^	−0.01	(−0.05, 0.02)	0.38	−0.02	(−0.05, 0.02)	0.37	−0.02	(−0.05, 0.02)	0.31
Change in DBP (mmHg)^*^	−0.003	(−0.04, 0.03)	0.86	−0.004	(−0.04, 0.03)	0.81	−0.01	(−0.04, 0.03)	0.74
BMI (kg/m^2^)	0.003	(−0.03, 0.04)	0.86	0.01	(−0.01, 0.03)	0.26	0.01	(−0.01, 0.03)	0.26
Change in BMI^*^(kg/m^2^)^*^	−0.02	(−0.06, 0.01)	0.16	−0.02	(−0.05, 0.01)	0.18	−0.02	(−0.05, 0.01)	0.18

	OR^$^	95%CI	*p*	OR^$^	95%CI	*p*	OR^$^	95%CI	*p*
Salt intake ≤ 6g/d	1.97	(1.45, 2.68)	< 0.01	1.96	(1.44, 2.66)	< 0.01	1.96	(1.44, 2.67)	< 0.01
Normal BP	1.01	(0.93, 1.28)	0.29	1.01	(0.93, 1.28)	0.29	1.01	(0.92, 1.29)	0.32

^#^Estimated based on linear regression; ^$^Estimated based on logistic regression; ^*^Calculated by subtracting baseline levels from 1 year after; ┼ Model A: crude model; ╫ Model B: adjusted for age, sex, alcohol usage and smoking, BMI; ╪Model C: adjusted for variables in Model B plus hypertension, diabetes, chronic gastritis, hyperglycemia and dyslipidemia.

SR, salt restriction; SS, salt substitute; eUNaE, estimated urinary sodium excretions; eUKE, estimated urinary potassium excretions; SBP, systolic blood pressure; DBP, diastolic blood pressure; BP, blood pressure; BMI, body mass index.

### Effects of SS on the level of eUKE and Na/K ratio

One year after salt intake restriction, the changes in eUKE levels in the two groups were opposite (*p* < 0.01), with the SR group showing a negative change (−0.40 (SD 0.64) g/day) and the SS group showing a positive change (0.01 (SD 0.46) g/day) (Table 2). Therefore, the SS group had a significantly higher level of eUKE after 1 year (2.09 (SD 0.43) g/day) than the SR group (1.71 (SD 0.62) g/day) (*p* < 0.01). Similarly, the Na/K ratio changed differently in the two groups (*p* < 0.01), with a positive change in the SR group [0.81 (SD 2.09)] and a negative change in the SS group [−0.38 (SD 1.00)]. The adjustment of confounding factors did not change the significance or direction of the association between SS and eUKE [*β* = 0.34, 95% (0.31, 0.37)], Na/K ratio [*β* = −0.33, 95% (−0.36, −0.30)], change in eUKE [*β* = 0.34, 95% (0.31, 0.37)] or change in Na/K ratio [*β* = −0.34, 95% (−0.37, −0.31)] (Table 3). However, despite consuming potassium-enriched salt substitute, only one participant in the SS group showed daily potassium intake ≥ 3.6 g, which was not significantly different from the SR group (*p* = 0.32) (Table 2). The effect of SS with 25% KCl on eUKE and Na/K ratio levels was slightly greater than that of SS with 13% KCl (*p* < 0.05) (Supplementary Tables S1, S2; Figure 2). The eUKE and Na/K ratio levels were similar between subjects exposed to SS for 9–11 and 12 months (*p* > 0.05) (Supplementary Tables S3, S4; Figure 2).

### Effects of SS on BP levels and BMI

Table 2 shows that after 1 year of restriction, both groups experienced an insignificant and subtle decrease in the levels of SBP, DBP, or BMI when compared to the baseline (*p* > 0.05). However, SR and SS showed no significant differences in SBP, DBP, BMI, or the risk of normal BP. The different dosages of KCl or duration of SS exposure were also not related to DBP or BMI. However, 25% KCl exposure was related to a lower level of SBP, regardless of whether it was compared with SR or 13% KCl (Supplementary Tables S1–S4; Figure 2).

### Sensitivity analyses

A sensitivity analysis was conducted in the unmatched overall cohort population, where SS had similar effects on eUNaE, eUKE, Na/K ratio, BP, and BMI, as observed in the PSM population (Figure 2; Supplementary Tables S5, S6). Stratification analysis showed that the effects of SS on BP, BMI, sodium, and potassium did not differ significantly between hypertensive and non-hypertensive subgroups (Supplementary Table S7).

### Safety outcomes

In the present study, we did not observe any AEs. Neither the SS nor SR groups showed severe arrhythmias (high apex of T waves, prolonged PR interval, multiple premature ventricular tachycardias, ventricular tachycardias, etc.) or hyperkalemia.

## Discussion

To the best of our knowledge, this study is the first to evaluate the impact of salt substitute on sodium and potassium intake, as well as blood pressure, among the general population in the real world. These findings indicate that salt restriction, including the consumption of salt-restricted spoons, has a very limited effect on improving sodium and potassium intake and that choosing salt substitute under the recommendation of physicians can significantly reduce sodium intake and increase potassium intake in the general population. However, the benefits of salt substitute in controlling blood pressure in the general population have not been shown, especially in the 13% KCl salt substitute group.

The expected impact of reducing sodium and increasing potassium intake can be calculated by replacing regular salt with a salt substitute. However, previous studies, including our own, have shown significant differences between theoretical predictions and actual measurements. In the SSaSS study, which was conducted among elderly participants in rural northern China, it was estimated that only 40–60% of dietary salt was replaced with salt substitute (19). In our own study, the SS group had a decrease of only − 0.68 g in sodium and an increase of 0.01 g in potassium. This may be due to an increase in sodium intake from processed foods worldwide as well as in China (20). Furthermore, our study was conducted among urban participants who consume more prepared food than rural residents. Additionally, the majority of salt substitutes available on the market contain only 13% KCl. Therefore, over 75% of participants in our study consumed salt substitute with 13% KCl. In contrast, the salt substitute used in the SSaSS and Yuan’s study contained 25% KCl provided by the researchers (5, 6). Therefore, our study may offer insight into the true effect of salt substitute on sodium and potassium intake among the general population in the real world.

Before the popularization of salt substitute, the primary strategy for minimizing salt consumption involved educating the public on salt restriction, particularly by promoting the use of salt-restricted spoons. Compared to salt restriction, exposure to salt substitute with either 13% or 25% KCl may result in lower levels of eUNaE, consistent with other study (21). The results indicate that salt substitute has a better effect in reducing eUNaE level than salt restriction for the public. The Chinese diet is well known as a high-sodium and low-potassium diet (4, 5, 22–24). Therefore, increasing potassium intake is equally important. Surprisingly, the level of eUKE decreased significantly in the salt restriction group after 1 year. Given that potassium intake primarily originates from fresh vegetables, we hypothesize that the intervention group’s consumption of high-potassium foods may decline. Regrettably, this study did not collect data on dietary intake and is thus unable to offer a definitive explanation for this observation. Furthermore, it leads to an increase in the Na/K ratio, which is associated with high blood pressure and cardiovascular disease (1, 2, 7). Therefore, in addition to sodium restriction, potassium addition should be generalized to the Chinese diet. Of course, based on the results in the current study, salt substitute may be an alternative strategy for elevating potassium intake and reducing the Na/K ratio.

Reducing sodium and/or increasing potassium intake has well-established blood pressure-lowering effects. There is empirical evidence that replacement of sodium substitute lowers systolic and diastolic blood pressure (average net changes [95% CI] were − 5.58[−7.08 to − 4.09] mmHg and − 2.88[−3.93 to − 1.83] mmHg, respectively) (25). Thus, salt substitute is a potential BP-lowering strategy under consideration by several countries, including China (26, 27). In our previous study, less sodium intake and higher potassium intake resulted in a decrease in blood pressure in hypertensive, normal blood pressure and hypotensive populations (28). Bruce et al. (5) observed a significant decrease in systolic blood pressure after implementation of the salt restriction strategy, and the change in blood pressure in the salt substitute group was greater than that in the routine salt restriction group. Yuan’s study suggested that the use of a salt substitute may lower blood pressure in elderly care facilities in China6. However, in our study, we did not observe significant changes in blood pressure. Nonetheless, we did find a difference in the change in SBP between the 25% KCl SS and SR groups, as well as between the 25% KCl SS and 13% KCl SS groups. Therefore, we believe that a low-level KCl salt substitute may not be as effective as a high-level salt substitute in reducing eUNaE and blood pressure. It’s noteworthy that the previous studies all utilized 25% KCl SS, which has beneficial effects on blood pressure reduction. However, in the area where this study was conducted, 13% KCl SS is currently more prevalent on the market. Based on our findings, we recommend promoting the production and distribution of 25% KCl SS alternatives. This approach could maximize the health benefits of sodium reduction while potentially enhancing potassium intake.

Moreover, we also found that even using 25% KCl as a salt substitute resulted in a lower reduction in systolic blood pressure compared to previous studies. The discrepancy may be because the SSaSS study enrolled individuals with high CVD risk factors4, whereas our group enrolled the general population with lower hypertension morbidity rates. A meta-analysis of 5 trials indicated that the beneficial effects of salt substitute on blood pressure was greater in participants with hypertension and smaller, nonsignificant in participants without hypertension 21. We conducted further analysis on the effect of salt substitute on blood pressure in the hypertensive population but found no significant changes. It is worth conducting further studies on the general population, especially those without hypertension or risk of cardiovascular disease.

The potential for an increased risk of hyperkalemia and related arrhythmia among participants using salt substitute was carefully evaluated in previous studies (4, 29–32). Overall, there is insufficient evidence regarding the effects of potassium-enriched salt substitute on the occurrence of hyperkalemia. Numerous cases of life-threatening hyperkalemia have been reported in chronic kidney disease or the use of medications that impair potassium excretion (33, 34). In the current study, all participants were asked by physicians to exclude contraindications; moreover, most of the participants used 13% KCl salt substitute in the current study. Thus, we did not find an adverse effect of salt substitute.

In summary, the current study first assessed the changes in urinary sodium and potassium levels, as well as blood pressure, by utilizing a salt substitute or implementing a salt restriction strategy at the population level in real-world settings in China. Our study has several limitations. First, this study did not use the gold standard 24-h urine test to evaluate sodium and potassium intake. Although the formula we employed is the least biased among the INTERSALT and Tanaka methods (35–37), potential bias might exist in our study, which may attenuate the observed association. Second, although the study adjusted for major sociodemographic characteristics and cardiometabolic factors, residual confounding in our study results may still exist. Third, an important limitation to consider is that the use of salt substitute was tracked only for home cooking, and we were unable to account for salt intake from meals consumed away from home. Fourth, we did not collect dietary questionnaires and distinguish between sodium and potassium intake from natural foods and those from additive sources. Finally, owing to the nature of a real-world propensity-matched retrospective cohort study, the current study was not equivalent to an RCT study, and the effect of salt substitute is worth further study.

## Conclusion

Compared with salt restriction, salt substitute over a year results in more sodium reduction and greater potassium increase, with no apparent adverse effects. The effect of salt substitute on lowering blood pressure in the general population requires further study in the real world.

## Data Availability

The raw data supporting the conclusions of this article will be made available by the authors, without undue reservation.
