# Supplementary material for: Effects of salt substitute on urinary electrolytes and blood pressure in a real-world setting—cohort study in Hunan, China
Source: Front Nutr. 2024 Dec 18;11:1504152. doi: 10.3389/fnut.2024.1504152 (PMC11688233; doi:10.3389/fnut.2024.1504152)
Supplement: Supplementary file 1 [file Table_1.docx]

| Table S1. Comparison of urinary sodium, potassium excretion, blood pressure, and BMI between SR group and two different SS dosage groups at one year after restriction implementation (N=3,794) | | | | | | | |
| --- | --- | --- | --- | --- | --- | --- | --- |
| Level of outcomes one year after the recommendation | SR (n=1,897) | SS with 13% KCL (n=1,480) | SS with 25% KCL (n=417) | *P* | *P*^╫^_SR Vs. 13%KCL_ | *P*^╫^_SR Vs. 25%KCL_ | *P*^╫^_13% Vs. 25%KCL_ |
| eUNaE (g/day), mean (SD) | 4.05(1.01) | 3.84(1.00) | 3.77(1.13) | <0.01 | <0.012 | <0.012 | 0.21 |
| eUKE (g/day), mean (SD) | 1.71(0.62) | 2.05(0.41) | 2.23(0.48) | <0.01 | <0.012 | <0.012 | <0.012 |
| Na/K ratio, mean (SD) | 3.02(2.12) | 1.94(0.77) | 1.73(0.63) | <0.01 | <0.012 | <0.012 | 0.02 |
| Change in eUNaE (g/day) *, mean (SD) | -0.42(1.16) | -0.59(0.85) | -1.02(1.45) | <0.01 | <0.012 | <0.012 | <0.012 |
| Change in eUKE (g/day) *, mean (SD) | -0.40(0.64) | -0.06(0.37) | 0.27(0.64) | <0.01 | <0.012 | <0.012 | <0.012 |
| Change in Na/K ratio*, mean (SD) | 0.81(2.09) | -0.21(0.70) | -1.01(1.52) | <0.01 | <0.012 | <0.012 | <0.012 |
| BMI (kg/m^2^), mean (SD) | 24.12(3.01) | 24.15(3.06) | 24.13(3.11) | 0.98 | 0.84 | 0.98 | 0.92 |
| Change in BMI (kg/m^2^) *, mean (SD) | 0.06(0.95) | 0.02(0.95) | 0.02(0.98) | 0.37 | 0.18 | 0.42 | 0.95 |
| SBP (mmHg), mean (SD) | 120.42(14.98) | 120.92(14.8) | 120.35(14.64) | 0.58 | 0.33 | 0.94 | 0.49 |
| DBP (mmHg), mean (SD) | 74.51(10.76) | 74.48(10.79) | 74.7(10.77) | 0.93 | 0.94 | 0.74 | 0.71 |
| Change in SBP (mmHg), mean (SD) | -0.18(11.42) | -0.21(11.3) | -1.54(11.39) | 0.08 | 0.93 | 0.03 | 0.04 |
| Change in DBP (mmHg), mean (SD) | 0.05(8.09) | 0.10(7.88) | -0.33(7.83) | 0.61 | 0.86 | 0.38 | 0.33 |
| Salt intake ≤6g/d, n (%) | 65(3.43) | 70(4.73) | 54(12.95) | <0.01 | 0.06 | <0.012 | <0.012 |
| Potassium intake ≥3.6g/d, n (%) | 0(0.00) | 1(0.07) | 0(0.00) | 0.46 | 0.26 | - | 0.60 |
| Normal BP, n (%) | 1541(81.23) | 1191(80.47) | 324(77.70) | 0.26 | 0.58 | 0.10 | 0.21 |
| * Calculated by subtracting baseline levels from one year after. ╫ difference between two groups was calculated by LSD test or the partition of chi square method (α′=α/((3*(3–1)/2)+1)=0.0125 was statistical significant)  Abbreviation: SR, salt restriction; SS, salt substitute; SD, standard deviance; eUNaE, estimated urinary sodium excretions; eUKE, estimated urinary potassium excretions; SBP, systolic blood pressure; DBP, diastolic blood pressure; BP, blood pressure. | | | | | | | |

| Table S2. Comparison of urinary sodium and potassium excretion, blood pressure, and BMI between SR group and two different SS exposure duration groups at one year after restriction implementation (N=3,794) | | | | | | | |
| --- | --- | --- | --- | --- | --- | --- | --- |
| Level of outcomes one year after the recommendation | SR (n=1,897) | SS used for 9-11 months  (n=264) | SS used for 12 months  (n=1,633) | *P* | *P*^╫^_SR Vs. SS 9-11 m_ | *P*^╫^_SR Vs. SS 12 m_ | *P*^╫^_SS 9-11 m % Vs. 12 m_ |
| eUNaE (g/day), mean (SD) | 4.05(1.01) | 3.88(1.14) | 3.81(1.01) | <0.01 | <0.012 | <0.012 | 0.35 |
| eUKE (g/day), mean (SD) | 1.71(0.62) | 2.09(0.48) | 2.09(0.42) | <0.01 | <0.012 | <0.012 | 0.90 |
| Na/K ratio, mean (SD) | 3.02(2.12) | 1.96(0.94) | 1.89(0.71) | <0.01 | <0.012 | <0.012 | 0.48 |
| Change in eUNaE (g/day) *, mean (SD) | -0.42(1.16) | -0.72(1.31) | -0.68(0.97) | <0.01 | <0.012 | <0.012 | 0.60 |
| Change in eUKE (g/day) *, mean (SD) | -0.40(0.64) | 0.001(0.58) | -0.02(0.44) | <0.01 | <0.012 | <0.012 | 0.70 |
| Change in Na/K ratio*, mean (SD) | 0.81(2.09) | -0.33(1.12) | -0.39(0.98) | <0.01 | <0.012 | <0.012 | 0.62 |
| BMI (kg/m^2^), mean (SD) | 24.12(3.01) | 24.18(3.22) | 24.14(3.04) | 0.96 | 0.78 | 0.91 | 0.83 |
| Change in BMI (kg/m^2^) *, mean (SD) | 0.06(0.95) | 0.06(0.91) | 0.01(0.96) | 0.28 | 0.96 | 0.12 | 0.46 |
| SBP (mmHg), mean (SD) | 120.42(14.98) | 119.91(14.79) | 120.94(14.54) | 0.43 | 0.60 | 0.30 | 0.30 |
| DBP (mmHg), mean (SD) | 74.51(10.76) | 74.28(10.58) | 74.57(10.82) | 0.92 | 0.75 | 0.87 | 0.69 |
| Change in SBP (mmHg), mean (SD) | -0.18(11.42) | -0.21(11.30) | -0.62(11.34) | 0.37 | 0.60 | 0.25 | 0.27 |
| Change in DBP (mmHg), mean (SD) | 0.05(8.09) | 0.30(7.83) | -0.04(7.88) | 0.80 | 0.64 | 0.73 | 0.52 |
| Salt intake ≤6g/d, n (%) | 65(3.43) | 24(9.09) | 100(6.12) | <0.01 | <0.012 | <0.012 | 0.07 |
| Potassium intake ≥3.6g/d, n (%) | 0(0.00) | 0(0.00) | 1(0.06) | 0.52 | - | 0.28 | 0.69 |
| Normal BP, n (%) | 1541(81.23) | 216 (81.82) | 1299(79.55) | 0.39 | 0.82 | 0.21 | 0.39 |
| * Calculated by subtracting baseline levels from one year after. ╫ difference between two groups was calculated by LSD test or the partition of chi square method (α′=α/((3*(3–1)/2)+1)=0.0125 was statistical significant)  Abbreviation: SR, salt restriction; SS, salt substitute; SD, standard deviance; eUNaE, estimated urinary sodium excretions; eUKE, estimated urinary potassium excretions; SBP, systolic blood pressure; DBP, diastolic blood pressure; BP, blood pressure. | | | | | | | |

| Table S3. The effect of exposure to two different salt substitutes dosage on urinary sodium and potassium excretion, blood pressure, and BMI compared to a regular salt restriction strategy after one year of restriction implementation | | | | | | |
| --- | --- | --- | --- | --- | --- | --- |
| Outcome at one year after restriction | SS with 13% KCL (n=1,480) | | | SS with 25% KCL (n=417) | | |
|  | β^╫^ | 95%CI | *P* | β^╫^ | 95%CI | *P* |
| eUNaE (g/day) | -0.21 | (-0.27, -0.14) | < 0.01 | -0.27 | (-0.37, -0.16) | < 0.01 |
| eUKE (g/day) | 0.60 | (0.54, 0.67) | < 0.01 | 0.92 | (0.82, 1.02) | < 0.01 |
| Na/K ratio | -0.64 | (-0.70, -0.57) | < 0.01 | -0.76 | (-0.86, -0.66) | < 0.01 |
| Change in eUNaE*(g/day) | -0.15 | (-0.22, -0.09) | < 0.01 | -0.55 | (-0.65, -0.44) | < 0.01 |
| Change in eUKE*(g/day) | 0.57 | (0.50, 0.63) | < 0.01 | 1.11 | (1.02, 1.21) | < 0.01 |
| Change in Na/K ratio*(g/day) | -0.58 | (-0.64, -0.52) | < 0.01 | -1.04 | (-1.14, -0.94) | < 0.01 |
| DBP (mmHg) | 0.01 | (-0.05, 0.07) | 0.78 | 0.02 | (-0.07, 0.12) | 0.62 |
| SBP (mmHg) | 0.04 | (-0.02, 0.10) | 0.17 | 0.002 | (-0.09, 0.09) | 0.96 |
| Change in SBP (mmHg) | -0.01 | (-0.08, 0.06) | 0.75 | -0.11 | (-0.21, -0.01) | 0.04 |
| Change in DBP (mmHg) | -0.0002 | (-0.07, 0.07) | 1.00 | -0.05 | (-0.15, 0.06) | 0.37 |
| BMI (kg/m^2^) | 0.02 | (-0.02, 0.05) | 0.33 | 0.02 | (-0.03, 0.08) | 0.41 |
| Change in BMI*(kg/m^2^) | -0.04 | (-0.11, 0.02) | 0.20 | -0.04 | (-0.15, 0.06) | 0.42 |
|  | OR^╫^ | 95%CI | *P* | OR^╫^ | 95%CI | *P* |
| Salt intake ≤6g/d | 1.39 | (0.99, 1.97) | 0.06 | 4.20 | (2.87, 6.16) | < 0.01 |
| Normal BP | 1.06 | (0.88, 1.27) | 0.53 | 1.20 | (0.91, 1.58) | 0.19 |
| * Calculated by subtracting baseline levels from one year after; ╫ adjusted for age, sex, alcohol usage and smoking, hypertension, diabetes, chronic gastritis, hyperglycemia, dyslipidemia;  Abbreviation: SR, salt restriction; SS, salt substitute; eUNaE, estimated urinary sodium excretions; eUKE, estimated urinary potassium excretions; SBP, systolic blood pressure; DBP, diastolic blood pressure; BP, blood pressure. | | | | | | |

| Table S4. The effect of different exposure duration of salt substitutes on urinary sodium and potassium excretion, blood pressure, and BMI compared to a regular salt restriction strategy after one year of restriction implementation | | | | | | |
| --- | --- | --- | --- | --- | --- | --- |
| Outcome at one year after restriction | SS used for 9-11 months (n=264) | | | SS used for 12 months (n=1,633) | | |
|  | β^╫^ | 95%CI | *P* | β^╫^ | 95%CI | *P* |
| eUNaE (g/day) | -0.17 | (-0.30, -0.04) | < 0.01 | -0.23 | (-0.30, -0.17) | < 0.01 |
| eUKE (g/day) | 0.38 | (0.31, 0.45) | < 0.01 | 0.38 | (0.35, 0.42) | < 0.01 |
| Na/K ratio | -1.05 | (-1.26, -0.85) | < 0.01 | -1.13 | (-1.23, -1.02) | < 0.01 |
| Change in eUNaE*(g/day) | -0.29 | (-0.43, -0.15) | < 0.01 | -0.26 | (-0.33, -0.19) | < 0.01 |
| Change in eUKE*(g/day) | 0.40 | (0.33, 0.47) | < 0.01 | 0.41 | (0.37, 0.45) | < 0.01 |
| Change in Na/K ratio*(g/day) | -1.15 | (-1.36, -0.94) | < 0.01 | -1.20 | (-1.30, -1.09) | < 0.01 |
| DBP (mmHg) | -0.14 | (-1.34, 1.06) | 0.82 | 0.17 | (-0.45, 0.78) | 0.59 |
| SBP (mmHg) | -0.29 | (-1.94, 1.36) | 0.73 | 0.61 | (-0.24, 1.46) | 0.16 |
| Change in SBP (mmHg) | 0.27 | (-1.17, 1.70) | 0.72 | -0.47 | (-1.21, 0.26) | 0.21 |
| Change in DBP (mmHg) | 0.15 | (-0.86, 1.16) | 0.77 | -0.12 | (-0.64, 0.40) | 0.64 |
| BMI (kg/m^2^) | 0.11 | (-0.09, 0.31) | 0.28 | 0.05 | (-0.05, 0.15) | 0.36 |
| Change in BMI*(kg/m^2^) | -0.003 | (-0.12, 0.12) | 0.96 | -0.05 | (-0.11, 0.02) | 0.14 |
|  | OR^╫^ | 95%CI | *P* | OR^╫^ | 95%CI | *P* |
| Salt intake ≤6g/d | 2.85 | (1.74, 4.66) | <0.01 | 1.83 | (1.33, 2.52) | <0.01 |
| Normal BP | 0.99 | (0.69, 1.40) | 0.93 | 1.11 | (0.93, 1.32) | 0.25 |
| * Calculated by subtracting baseline levels from one year after; ╫ adjusted for age, sex, alcohol usage and smoking, hypertension, diabetes, chronic gastritis, hyperglycemia, dyslipidemia;  Abbreviation: SR, salt restriction; SS, salt substitute; eUNaE, estimated urinary sodium excretions; eUKE, estimated urinary potassium excretions; SBP, systolic blood pressure; DBP, diastolic blood pressure; BP, blood pressure. | | | | | | |

| Table S5. Comparison of urine sodium, potassium excretion, blood pressure, and BMI between different restriction strategy groups at one year after restriction implementation among unmatched overall cohort (N=5,000) | | | |
| --- | --- | --- | --- |
| Level of outcomes at one year after restriction | SR (n=2,929) | SS (n=2,071) | *P* |
| eUNaE (g/day), mean (SD) | 4.43 (1.08) | 4.51 (1.01) | < 0.01 |
| eUKE (g/day), mean (SD) | 2.27 (0.47) | 1.98 (0.45) | < 0.01 |
| Na/K ratio, mean (SD) | 2.86 (2.04) | 1.97 (0.93) | < 0.01 |
| Change in eUNaE (g/day) *, mean (SD) | -0.48 (1.16) | -0.77 (1.02) | < 0.01 |
| Change in eUKE (g/day) *, mean (SD) | -0.54 (0.67) | 0.04 (0.55) | < 0.01 |
| Change in Na/K ratio*, mean (SD) | 0.75 (1.96) | -0.67 (1.39) | < 0.01 |
| BMI (kg/m^2^), mean (SD) | 24.45 (3.02) | 24.15 (3.09) | < 0.01 |
| Change in BMI (kg/m^2^) *, mean (SD) | 0.05 (0.96) | 0.02 (0.92) | 0.20 |
| SBP (mmHg), mean (SD) | 120.80 (14.68) | 120.66 (14.79) | 0.74 |
| DBP (mmHg), mean (SD) | 74.99 (10.66) | 74.48 (10.82) | 0.09 |
| Change in SBP (mmHg), mean (SD) | -0.10 (11.43) | -0.46 (11.41) | 0.27 |
| Change in DBP (mmHg), mean (SD) | -0.004 (8.13) | -0.03 (7.96) | 0.90 |
| Salt intake ≤6g/d, n (%) | 109 (3.72) | 133 (6.42) | < 0.01 |
| Potassium intake ≥3.6g/d, n (%) | 0 (0.00) | 1 (0.05) | 0.23 |
| Normal BP, n (%) | 2397 (81.84) | 1658 (80.06) | 0.11 |
| * Calculated by subtracting baseline levels from one year after.  Abbreviation: SR, salt restriction; SS, salt substitute; SD, standard deviance; eUNaE, estimated urinary sodium excretions; eUKE, estimated urinary potassium excretions; SBP, systolic blood pressure; DBP, diastolic blood pressure; BP, blood pressure; BMI, body mass index. | | | |

| Table S6. Effects of salt substitute usage on urinary sodium and potassium excretion, blood pressure, and BMI compared to a regular salt restriction strategy after one year of restriction implementation among unmatched overall cohort (N=5,000) | | | |
| --- | --- | --- | --- |
| Outcome at one year after restriction | Effect coefficient | | |
|  | β^╫^ | 95%CI | *P* |
| eUNaE (g/day) | -0.11 | (-0.14, -0.08) | < 0.01 |
| eUKE (g/day) | 0.16 | (0.14, 0.19) | < 0.01 |
| Na/K ratio | -0.24 | (-0.26, -0.21) | < 0.01 |
| Change in eUNaE*(g/day) | -0.11 | (-0.13, -0.09) | < 0.01 |
| Change in eUKE*(g/day) | 0.40 | (0.37, 0.42) | < 0.01 |
| Change in Na/K ratio*(g/day) | -0.35 | (-0.37, -0.33) | < 0.01 |
| DBP (mmHg) | 0.01 | (-0.02, 0.03) | 0.47 |
| SBP (mmHg) | -0.29 | (-0.003, 0.05) | 0.09 |
| Change in SBP (mmHg) | -0.02 | (-0.05, 0.01) | 0.14 |
| Change in DBP (mmHg) | -0.01 | (-0.03, 0.02) | 0.59 |
| BMI (kg/m^2^) | 0.003 | (-0.01, 0.02) | 0.63 |
| Change in BMI*(kg/m^2^) | -0.02 | (-0.04, 0.01) | 0.25 |
|  | OR^╫^ | 95%CI | *P* |
| Salt intake ≤6g/d | 1.72 | (1.32, 2.23) | <0.01 |
| Normal BP | 1.14 | (0.98, 1.32) | 0.10 |
| * Calculated by subtracting baseline levels from one year after; ╫ adjusted for age, sex, alcohol usage and smoking, hypertension, diabetes, chronic gastritis, hyperglycemia, dyslipidemia;  Abbreviation: SR, salt restriction; SS, salt substitute; eUNaE, estimated urinary sodium excretions; eUKE, estimated urinary potassium excretions; SBP, systolic blood pressure; DBP, diastolic blood pressure; BP, blood pressure. | | | |

| Table S7. Effects of salt substitute usage compared to regular salt restriction strategy by hypertension status (N=3,794) | | | | | | | | | |
| --- | --- | --- | --- | --- | --- | --- | --- | --- | --- |
| Outcome at one year after restriction | | Effect coefficient | | | Outcome at one year after restriction | | Effect coefficient | | |
|  | | β^╫^ | 95%CI | *P* |  | | β^╫^ | 95%CI | *P* |
| eUNaE (g/day) | | |  |  | Change in eUNaE*(g/day) | | |  |  |
|  | Hypertension | -0.12 | (-0.20, -0.04) | < 0.01 |  | Hypertension | -0.14 | (-0.22, -0.05) | < 0.01 |
|  | No hypertension | -0.11 | (-0.14, -0.07) | < 0.01 |  | No hypertension | -0.12 | (-0.15, -0.08) | < 0.01 |
|  | *P* for interaction |  |  | 0.80 |  | *P* for interaction |  |  | 0.69 |
| eUKE (g/day) | |  |  |  | Change in eUKE*(g/day) | | |  |  |
|  | Hypertension | 0.32 | (0.25, 0.40) | < 0.01 |  | Hypertension | 0.33 | (0.25, 0.40) | < 0.01 |
|  | No hypertension | 0.34 | (0.31, 0.37) | < 0.01 |  | No hypertension | 0.35 | (0.32, 0.38) | < 0.01 |
|  | *P* for interaction |  |  | 0.52 |  | *P* for interaction |  |  | 0.43 |
| Na/K ratio | |  |  |  | Change in Na/K ratio*(g/day) | | |  |  |
|  | Hypertension | -0.32 | (-0.40, -0.24) | < 0.01 |  | Hypertension | -0.33 | (-0.41, -0.25) | < 0.01 |
|  | No hypertension | -0.34 | (-0.37, -0.30) | < 0.01 |  | No hypertension | -0.34 | (-0.38, -0.31) | < 0.01 |
|  |  |  |  | 0.47 |  | *P* for interaction |  |  | 0.48 |
| DBP (mmHg) | |  |  |  | Change in DBP (mmHg) | | |  |  |
|  | Hypertension | 0.11 | (-0.07, 0.10) | 0.80 |  | Hypertension | -0.04 | (-0.14, 0.05) | 0.40 |
|  | No hypertension | 0.004 | (-0.03, 0.03) | 0.78 |  | No hypertension | -0.001 | (-0.03, 0.03) | 0.95 |
|  | *P* for interaction |  |  | 0.82 |  | *P* for interaction |  |  | 0.51 |
| SBP (mmHg) | |  |  |  | Change in SBP (mmHg) | | |  |  |
|  | Hypertension | 0.01 | (-0.08, 0.09) | 0.86 |  | Hypertension | -0.05 | (-0.16, 0.05) | 0.33 |
|  | No hypertension | 0.01 | (-0.01, 0.04) | 0.32 |  | No hypertension | -0.01 | (-0.05, 0.02) | 0.42 |
|  | *P* for interaction |  |  | 0.70 |  | *P* for interaction | 0.61 |  |  |
| BMI (kg/m^2^) | |  |  |  | Change in BMI*(kg/m^2^) | | |  |  |
|  | Hypertension | 0.002 | (-0.04, 0.05) | 0.93 |  | Hypertension | -0.06 | (-0.14, 0.02) | 0.17 |
|  | No hypertension | 0.01 | (-0.01, 0.03) | 0.26 |  | No hypertension | -0.02 | (-0.05, 0.02) | 0.36 |
|  | *P* for interaction |  |  | 0.85 |  | *P* for interaction |  |  | 0.44 |
|  |  | OR^╫^ | 95%CI | *P* |  |  | OR^╫^ | 95%CI | *P* |
| Salt intake ≤6g/d | |  |  |  | Normal BP | |  |  |  |
|  | Hypertension | 2.97 | (1.19, 7.40) | 0.02 |  | Hypertension | 1.29 | (0.91, 1.83) | 0.15 |
|  | No hypertension | 1.85 | (1.33, 2.57) | < 0.01 |  | No hypertension | 1.02 | (0.83, 1.24) | 0.88 |
|  | *P* for interaction |  |  | 0.37 |  | *P* for interaction |  |  | 0.22 |
| * Calculated by subtracting baseline levels from one year after. ╫ adjusted for age, sex, alcohol usage and smoking, hypertension, diabetes, chronic gastritis, hyperglycemia, dyslipidemia;  Abbreviation: SR, salt restriction; SS, salt substitute; SD, standard deviance; eUNaE, estimated urinary sodium excretions; eUKE, estimated urinary potassium excretions; SBP, systolic blood pressure; DBP, diastolic blood pressure; BP, blood pressure; BMI, body mass index. | | | | | | | | | |
